# A patient-like swine model of gastrointestinal fibrotic strictures for advancing therapeutics

**DOI:** 10.1038/s41598-021-92628-8

**Published:** 2021-06-25

**Authors:** Ling Li, Mohamad I. Itani, Kevan J. Salimian, Yue Li, Olaya Brewer Gutierrez, Haijie Hu, George Fayad, Jean A. Donet, Min Kyung Joo, Laura M. Ensign, Vivek Kumbhari, Florin M. Selaru

**Affiliations:** 1 Department of Medicine and Division of Gastroenterology and Hepatology, Johns Hopkins Medical Institutions, 720 Rutland Avenue, Ross Building, Room 950, Baltimore, MD 21205 USA; 2 Department of Pathology, Johns Hopkins Medical Institutions, Baltimore, MD USA; 3 Department of Ophthalmology, The Center for Nanomedicine At the Wilmer Eye Institute, Johns Hopkins Medical Institutions, Baltimore, MD USA; 4 Departments of Chemical and Biomolecular Engineering, Biomedical Engineering, Pharmacology and Molecular Sciences, Gynecology and Obstetrics, Infectious Diseases, and Oncology, Johns Hopkins University, Baltimore, MD USA

**Keywords:** Gastroenterology, Oesophagogastroscopy

## Abstract

Gastrointestinal (GI) strictures are difficult to treat in a variety of disease processes. Currently, there are no Food and Drug Administration (FDA) approved drugs for fibrosis in the GI tract. One of the limitations to developing anti-fibrotic drugs has been the lack of a reproducible, relatively inexpensive, large animal model of fibrosis-driven luminal stricture. This study aimed to evaluate the feasibility of creating a model of luminal GI tract strictures. Argon plasma coagulation (APC) was applied circumferentially in porcine esophagi in vivo. Follow-up endoscopy (EGD) was performed at day 14 after the APC procedure. We noted high grade, benign esophageal strictures (n = 8). All 8 strictures resembled luminal GI fibrotic strictures in humans. These strictures were characterized, and then successfully dilated. A repeat EGD was performed at day 28 after the APC procedure and found evidence of recurrent, high grade, fibrotic, strictures at all 8 locations in all pigs. Pigs were sacrificed and gross and histologic analyses performed. Histologic examination showed extensive fibrosis, with significant collagen deposition in the lamina propria and submucosa, as well as extensive inflammatory infiltrates within the strictures. In conclusion, we report a porcine model of luminal GI fibrotic stricture that has the potential to assist with developing novel anti-fibrotic therapies as well as endoscopic techniques to address recurring fibrotic strictures in humans.

## Introduction

A fibrotic stricture can occur at several locations in the GI tract, such as the esophagus (peptic disease, eosinophilic esophagitis, post-endoscopic mucosal resection, post-submucosal dissection, anastomotic after bowel surgery, after caustics ingestion)^1^, small bowel (Crohn’s disease, CD), colon (CD^2^, ulcerative colitis (UC)^3^, ischemic, post-diverticulitis), biliary tree (anastomotic stricture after liver transplant or surgery to the biliary tree^4–6^) and other locations. Current endoscopic management of GI strictures include bougie or balloon dilation, stent placement, intra-stricture steroid injections, and needle-knife stricturotomy^7,8^. Unfortunately, most strictures recur, requiring frequent and in some cases life-long endoscopic procedures. In many patients, despite aggressive frequent dilation, salvage surgical resection or stricturoplasty is necessary, often associated with considerable morbidity and mortality^9^.

Some of the unifying features of GI luminal strictures across disease processes are (1) extracellular collagen deposition and fibrosis, and (2) our current inability to prevent recurrence after endoscopic/surgical treatment^10^. For example, in Crohn’s disease, the prevailing view has traditionally been that fibrosis could be effectively treated or prevented by treating the inflammation. However, the past 20 years of clinical experience treating Crohn’s patients with potent anti-inflammatory biologics (anti-TNF-α, anti-IL-12/23, etc.) has revealed little to no impact on the progression of fibrostenosing complications in Crohn’s patients^11^. One of the limitations to advancing anti-fibrotics to date has been the lack of a large animal model of fibrosis-driven luminal stricture. There is an immediate need to develop such a model to advance the development of novel therapies using standard commercially available endoscopic equipment. Due to its ease of access, straightforward anatomical configuration, and absence of the need for specialized preparation, the esophagus is a logical location for such a model. Pigs are the ideal species for the creation of a large animal model given that their GI anatomy closely resembles that of humans, also because of their accessibility and cost, and the endoscopists’ familiarity.

Endoscopic submucosal dissection (ESD) and endoscopic mucosal resection (EMR) have been used to create large animal esophageal stricture models in the past^12,13^. The two approaches, however, are technically complex, time consuming, and are often marred by a high rate of complications^14–16^. Other methods such as endoscopic radiofrequency ablation (RFA), it is technically quite simple, however the stricture created are short and not durable^17^. Another method of cap ligation followed by mucosal inversion and snare resection was effective at creating esophageal stricture, require a double channel endoscope^18,19^. Therefore, there is a necessity to explore a simple, safe and reproducible approach to create pre-clinical models to promote the development of therapies.

Argon plasma coagulation (APC) is a non-contact thermal method commonly used to treat gastrointestinal bleeding as well as to de-bulk tumors in cases where surgery is not recommended^20,21^. Compared with ESD, APC is less technically demanding, less time-consuming, and has a superior safety profile. The aim of this study was to create a large animal model of luminal GI strictures that can demonstrate a strong fibrotic component, as well as tendency to recur after endoscopic treatment.

## Results

### First EGD with APC mucosal ablation

**Figure 1:**
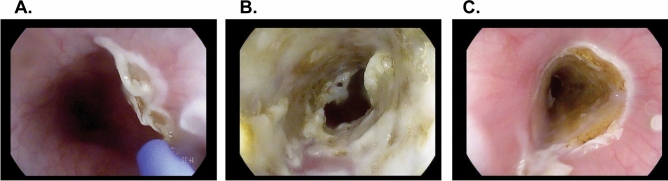
Endoscopic appearance during circumferential esophageal APC. (**A**) APC induced stricture procedure. The ablation can be performed either from proximal to distal or distal to proximal. (**B**) Appearance after APC ablation. Note the white/golden tissue effect. (**C**) Post-ablation appearance at 30–33 cm. Note the homogenous and circumferential aspect of ablated site.

APC was successfully performed at a total of 8 different locations in the esophagi of 3 pigs (Supplementary Fig. 1). The sites of mucosal ablation were at 30, 40 and 50 cm from the incisors. All procedures were successful in achieving circumferential ablation (as appreciated by a continuous layer of white/golden tissue) to the esophageal mucosa at the electrosurgical settings shown in Supplementary Table 1. The ablated surface was 1 cm in length in one location and 3–5 cm in length in all others. The average APC time was 15 min per location. There were no technical difficulties or complications such as bleeding or perforation noted. Figure 1 shows the endoscopic appearance of APC-induced ablation. Mucosal effect was documented as a homogenous whitish-yellowish discoloration of the ablated mucosa.

### Post-procedure follow-up and surveillance

All pigs recovered well after the index EGD with APC. There were no immediate adverse events (AEs) such as fever, pain, bleeding, or perforation. All pigs were able to tolerate a liquid diet on day 1 and a regular/solid diet thereafter. The amount of solid food intake was unrestricted when provided.

### Second EGD with stricture dilation (day 14)

**Figure 2:**
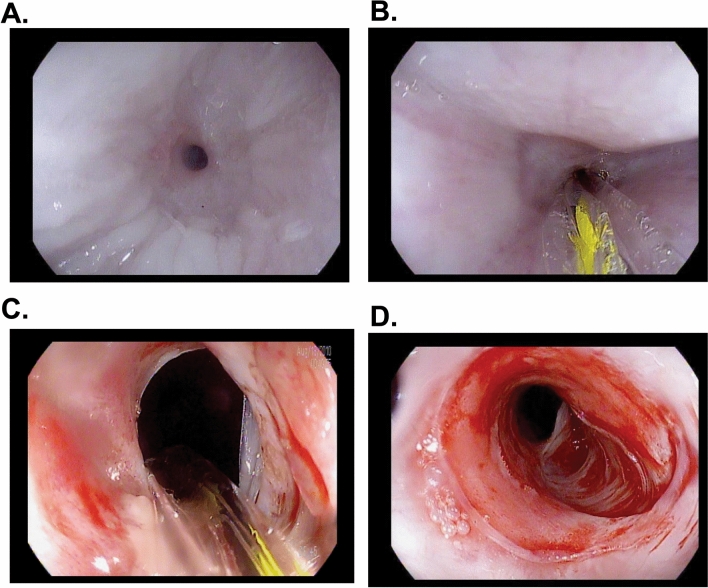
Endoscopic appearance of the esophageal stricture pre and post dilation at Day 14. (**A**) Fibrotic esophageal stricture with minimal residual inflammation measuring approximately 3 mm in diameter. (**B**) A 0.035 inch guidewire was advanced through the stricture followed by insertion of a wire guided through-the-scope (TTS) balloon for dilation. (**C**) Appearance after dilation to 9 mm. The deflated balloon and guidewire remains in place. (**D**) Stricture appearance post dilation. Note the tear exposing the submucosal fibers at the 4 o’clock position.

All pigs underwent endoscopic follow-up and balloon dilation at day 14 (Fig. 2). Strictures were present at all ablated sites (two in pig 1, three in pigs 2 and 3) (Supplementary Table 1). The luminal diameter at the stricture site was 1 to 6 mm, with an average of 3.25 mm. All strictures were successfully dilated to 9 mm. There was mild and self-limited oozing, as expected from our experience in patients, with no required endoscopic treatment.

### Systemic symptoms of GI obstruction

In the first 4–7 days after the first follow-up endoscopy with stricture dilation, two pigs exhibited systemic symptoms. Pig number 2 developed progressive solid food intolerance characterized by vomiting and regurgitation, which started at day 18 (4 days after stricture dilation). Diet was downgraded to liquids. The pig survived until day 28 on liquid diet. Pig number 3 developed progressive weight loss, but no vomiting or regurgitation. This pig was successfully maintained on regular diet. Pig number 1 had no symptoms of luminal obstruction and also did not experience weight loss (Supplementary Table 1).

### Third EGD with fluoroscopy (day 28)

**Figure 3:**
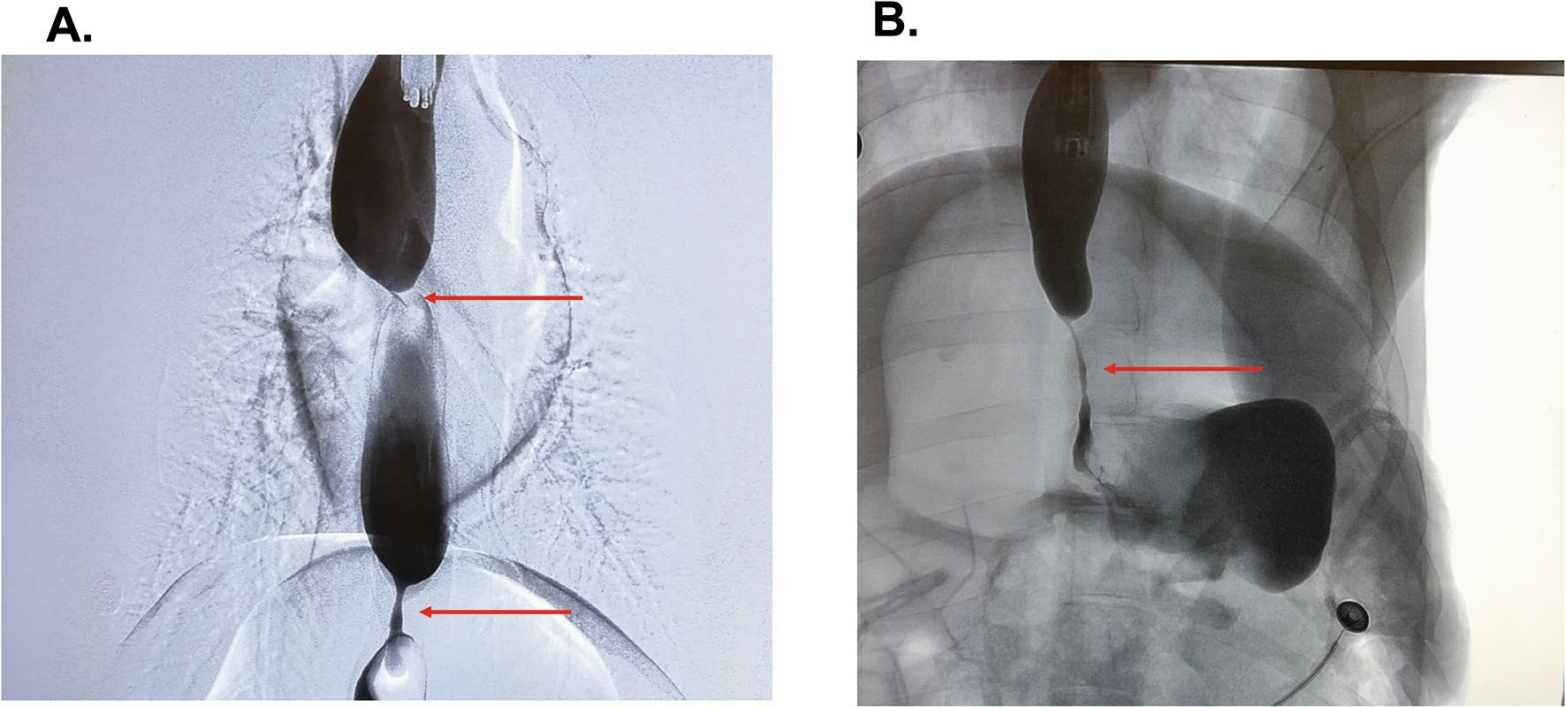
Fluoroscopy at day 28 showing proximal and mid esophageal strictures. (**A**) Contrast injection from the proximal esophagus revealed two strictures. There was a 1 cm stricture at 30 cm (proximal, arrow) and another 3 cm stricture at 40 cm (mid, arrow) from the incisors. Note the upstream dilation of the esophagus as a result of the strictures. (**B**) Contrast injection revealing a uniform 5 cm distal esophageal stricture at 50 cm from the incisors. The diameter of the stricture was 0.5–1 mm. Note the contrast in the gastric fundus downstream from the stricture indicating that the stricture is not causing complete luminal obstruction.

**Figure 4:**
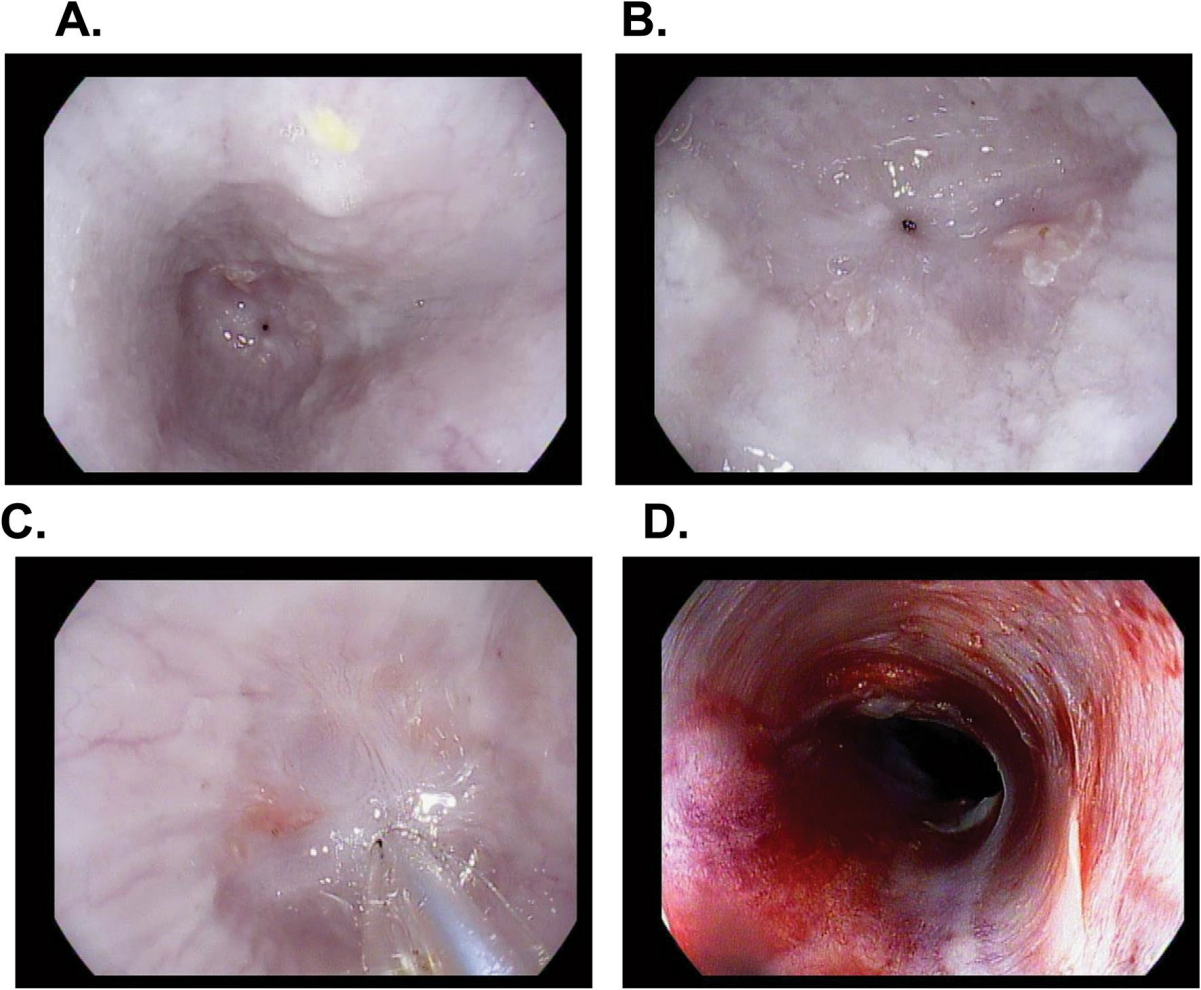
Endoscopic appearance of the esophageal strictures at day 28 (14 days after balloon dilation to 9 mm). (**A**) Appearance of a pinhole stricture (1 mm in diameter) with upstream dilation of the esophagus. (**B**) Appearance of the esophagus 2 cm proximal to the stricture. (**C**) The TTS balloon was advanced through the stricture and ready for dilation. (**D**) Appearance of the stricture after dilation to 9 mm. Note the tear encompassing 75% of the circumference of the esophageal lumen.

**Figure 5:**
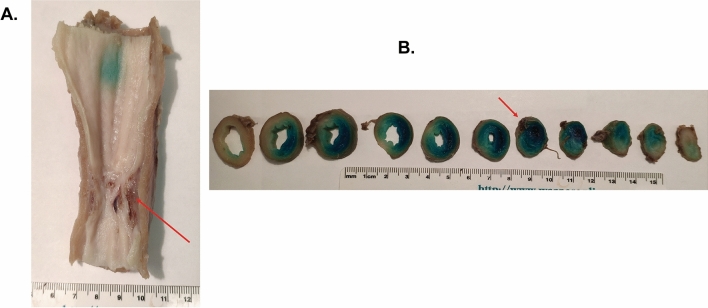
Gross appearance of the swine esophageal stricture at day 28. (**A**) Longitudinal segment of the esophagus with the stricture (arrow). (**B**) Serial cross sections from proximal to distal (left to right). The lumen progressively narrows to a point of near complete obstruction (arrow).

The third and last endoscopy was performed 14 days after the second EGD with stricture dilation (day 28 from index EGD with APC ablation). It was noted that all strictures re-stenosed and that the degree of stenosis was worse at all sites in all 3 pigs. The average lumen diameter was 1.9 mm, despite successful balloon dilation to 9 mm at day 14 (Supplementary Table 1). Fluoroscopic findings were recorded (Fig. 3). Pigs 2 and 3 exhibited tight luminal strictures at 50 cm, as shown in Figs. 3 and 4. There was one stricture (Pig 3 lesion 3c) in which the 0.035 inch guidewire was unable to traverse (diameter < 0.5 mm), and hence, balloon dilation was not performed (Supplementary Table 1. Such a high grade stricture in humans would have necessitated surgical intervention. Of note, pig 2 developed clinical symptoms of luminal stricture 4 days after dilation at day 14 (regurgitation and weight loss) (Supplementary Table 1). The clinical symptoms as well as radiologic and endoscopic appearance at day 28 indicate that stricture re-formation developed soon after dilation at day 14. Figure 5 shows the balloon dilation technique of a 1 mm-diameter stricture, with visible post-dilation mucosal and submucosal tearing and mild oozing.

### Gross and histopathologic findings

**Figure 6:**
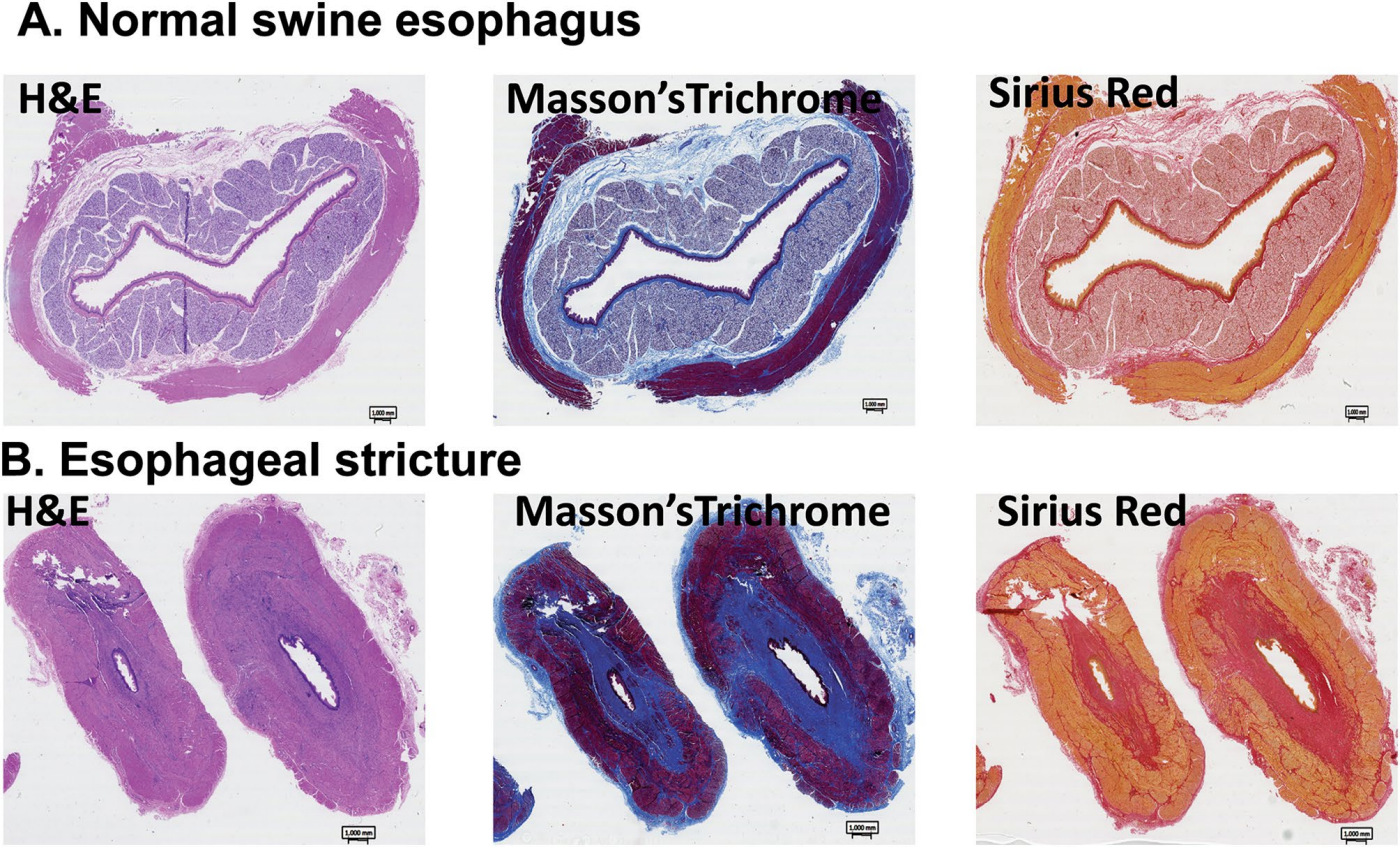
Histologic evidence of fibrosis at site of stricture formation. (**A**) Cross section of esophagus showing normal anatomy. Histologic evaluation shows no inflammatory changes or fibrosis. (**B**) Cross section of APC-induced esophageal stricture showing advanced fibrosis. H&E staining shows significant fibrosis of the lamina propria and submucosa (confirmed by Masson’s trichrome and Sirius Red). Note the significant narrowing of the lumen caused by APC-induced fibrosis.

**Figure 7:**
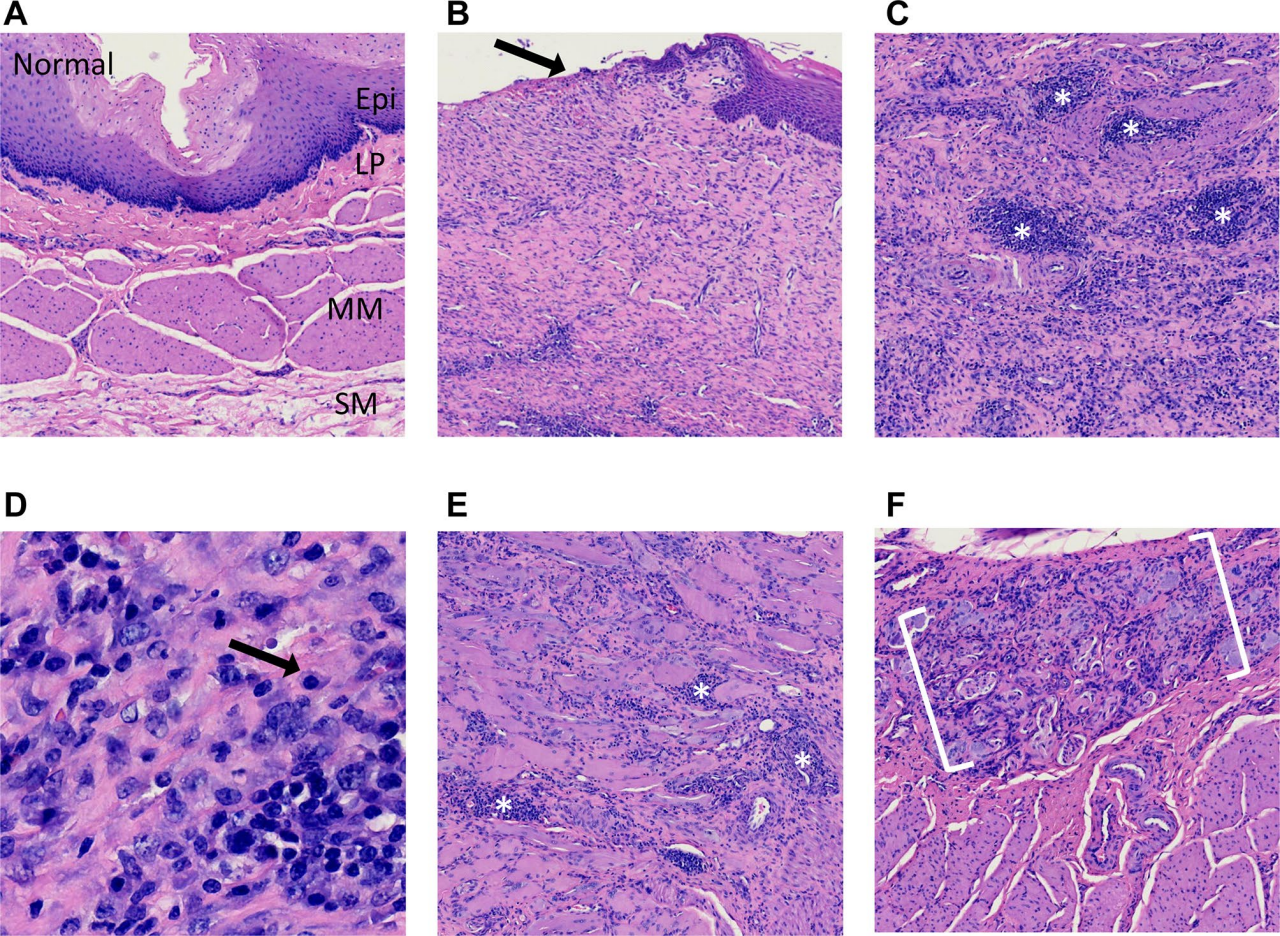
Histopathologic and inflammatory changes present at site of stricture formation. (**A**) Section from untreated, non-strictured esophagus showing normal anatomy with no fibrosis or inflammation. Original magnification: 4×. (**B**) APC-treated, strictured esophagus shows erosion of epithelium (arrow) and dense underlying fibrosis of lamina propria and submucosa. Original magnification: 4×. (**C**) Dense chronic inflammatory infiltrate present within the fibrotic submucosa. Asterisks denote lymphoid aggregates. Original magnification: 10×. (**D**) High magnification view of the chronic inflammatory infiltrate which is composed of a mixture of B- and T-cells and occasional plasma cells (arrow). Original magnification: 25×. (**E**) Lymphoid aggregates (asterisks) are present in the superficial muscularis propria. Original magnification: 4×. (**F**) Prominent neural hyperplasia (brackets) is present within the muscularis propria. Abbreviations: Epi = epithelium; LP = lamina propria; MM = muscularis mucosae; SM = submucosa.

At day 28, after the second follow-up endoscopy, all pigs were euthanized. Gross images of the strictured esophagi are displayed in Fig. 5. Serial cross sectioning at the time of harvest revealed near complete obstruction of the esophageal lumen in one case (Fig. 5B). To assess the microscopic pattern of injury and degree of fibrosis as well as inflammation following APC ablation, we used hematoxylin and eosin, Masson’s trichrome and Sirius Red staining on sections of APC-treated and untreated esophagi (Fig. 6). Histologic examination with higher magnification of the untreated esophagi revealed completely normal anatomy (i.e. normal squamous epithelium with no appreciable underlying inflammation or fibrosis of the lamina propria, submucosa or muscularis layer, Fig. 7A). On the contrary, at all sites of stricture formation in the APC-treated esophagi, histologic examination revealed reactive squamous epithelial changes with foci of erosion and dense underlying extensive lamina propria and submucosal fibrosis (Fig. 7B). The muscularis propria had minimal superficial fibrosis. Present within the fibrotic lamina propria and submucosa was a chronic inflammatory infiltrate composed of a mixture of B- and T-cells, occasional plasma cells and scattered neutrophils with interspersed lymphoid aggregates (Fig. 7C, D). While the chronic inflammatory infiltrate was largely restricted to the lamina propria and submucosa, occasional foci of lymphoid aggregates were identified in areas of the superficial muscularis propria that had undergone fibrosis (Fig. 7E). Also notable within the muscularis propria was the presence of prominent neural hyperplasia (Fig. 7F).

## Discussion

Gastrointestinal (GI) strictures represent an abnormal narrowing of the GI lumen. These are commonly seen in clinical practice and pose a therapeutic challenge. There are multiple etiologies for strictures, such as chronic inflammation in CD and UC^22^, caustic ingestion, and complications of resection in the treatment of GI cancer and Barrett’s esophagus^7,23,24^, among others. To date, there are no FDA-approved anti-fibrotic drugs to delay or reverse stricture formation. Endoscopic therapy including bougie/balloon dilation, pneumatic dilation, stenting, and surgery have been the mainstay of treatment. Endoscopic dilation, however, provides only a temporary relief, with a large proportion of patients requiring repeated and frequent dilations with an associated increased risk of complications^25–27^. Intra-lesion injection of steroids or mitomycin C have been used at the time of dilation^7,24,28–34^, however, outcomes have not justified a recommendation to use these routinely.

A multitude of approaches have been suggested to prevent stricture formation or progression, however, a definitive preventative therapy does not yet exist^7,24,28–34^. The development of new endoscopic accessories and therapeutic agents is hindered by the lack of a suitable large animal model of GI strictures. An ideal model should be a large animal, relatively inexpensive, technically feasible, and safe. In addition, an ideal model of luminal GI stricture should demonstrate a dense fibrotic reaction and stricture re-formation after balloon dilation (similar to human fibrotic GI strictures). ESD has been used to create swine esophageal strictures for in vivo pre-clinical studies^35,36^. In this study, we report a novel technique using APC to create an *in-vivo* pig esophageal stricture model for therapeutic studies. Compared to ESD, APC is technically easier, is less time consuming and requires significantly less expertise. Therefore, we suggest that the method described by us is ideal for the purpose of a large animal model of fibrotic luminal GI strictures.

In this study, we created 8 strictures in a total of 3 pigs using APC. In follow-up endoscopies at days 14 and 28, we successfully demonstrated persistence of 8 out of 8 APC-induced strictures. CRE balloon dilation was also feasible in all but one stricture (due to intense fibrosis and high grade stricture). No major complications from stricture creation was noted. We also demonstrated that all strictures recurred and worsened within two weeks post dilation. It is important to note that this model of swine stricture recurrence reflects our clinical experience in patients. Fluoroscopy confirmed the presence and re-formation of all strictures. Additionally, histologic examination showed fibrosis of the lamina propria and submucosa at the strictured sites.

Luminal GI strictures with a strong fibrotic component can reliably be modeled in swine using APC. The protocol outlined here resulted in fibrotic strictures that are relevant to human diseases, including peptic esophageal strictures, post-surgical anastomotic strictures and some of the inflammatory bowel disease-related strictures. The presence of inflammation at site of strictures mirrors a similar scenario seen in strictures in IBD (CD and UC). Mirroring typical behavior of human luminal GI strictures, the created strictures demonstrated a strong and recurrent fibrotic component refractory to endoscopic dilation. The demonstration of induction of fibrosis, inflammation associated with the strictures, and particularly natural recurrence of strictures in this model after endoscopic dilation, render this novel swine model of GI strictures unique. The technique we used was feasible, effective, safe, reproducible, and relatively inexpensive. The swine model of luminal GI fibrotic stricture reported in this study is the first of its kind and has the potential to assist in developing novel anti-fibrotic therapies, as well as creating endoscopic and surgical techniques to address these fibrotic recurring strictures in humans.

## Methods

### Animals

Three heathy Yorkshire pigs (*Sus scrofa domestica, female, 35–40 kg)* were purchased (Archer Farms, Darlington, MD) and housed in the Johns Hopkins large animal facility. Pigs were fed with a commercial porcine diet (NO. 8753, Harlan Tekland, Madison, WI), and water was provided ad libitum. After arrival, pigs were acclimated for 1 week ahead of procedure as described^37^. This study was performed under the approval of the Johns Hopkins University Animal Care and Use Committee (ACUC). All procedures were conducted in compliance with the Guide for the Care and Use of Laboratory Animals, as well as the Animal Welfare Act, applicable Animal Welfare Regulations at an AAALAC-accredited facility^38–40^. Furthermore, the study was carried out in compliance with the Animal Research: Reporting of in Vivo experiments (ARRIVE) guidelines^41^.

### Anesthesia

Pigs were fasted overnight. Pigs were sedated with intramuscular injection of a cocktail made of 1 mg/kg Telazol reconstituted with 25 mg/kg Ketamine and 2.5 mg/kg xylazine-(TKX). Pigs were transferred to the procedure room, intubated with a 7.5 French endotracheal tube and general anesthesia was maintained with 0.4–0.6% Isoflurane gas (VetOne, Boise, Idaho). 0.9% Sodium Chloride was administered via an intravenous line in a marginal ear vein. Heart rate, respiratory rate, blood pressure, temperature, end-tidal CO_2_, oxygen saturation (SPO_2_), and electrocardiogram (ECG) were monitored by a veterinary technician anesthetist throughout the procedures and were recorded each 15 min. Pigs were placed in a supine position for endoscopy. Following the endoscopy, pigs were returned to the animal facility after they recovered from sedation.

### Argon plasma coagulation (APC) procedure

All procedures were performed using a forward-viewing single channel upper gastrointestinal endoscope and a video scope system (EG29-i10 Video Gastroscope Standard HD+ , PENTAX Medical, USA). The endoscope was inserted through the mouth to the esophagus and stomach. An initial complete endoscopic examination of the esophageal and gastric mucosa was performed to ensure there were no pre-existing lesions. APC was carried out with an electrosurgical device system, an APC generator (VIO 300D) and an APC2-unit (both ERBE, Tübingen, Germany). The electrical setting was pulsed APC, effect 2, 30–40 Watts. The argon flow was set at 0.8 L/min. APC was applied in a circumferential manner using a straight fire forward catheter, creating a 3 cm longitudinal area of mucosal devitalization (Supplementary Table 1). APC was performed until the mucosa was white to golden in color, indicating adequate mucosal damage. Two APC ablations were performed in the first pig. We noted that there was room for an additional ablation. Therefore, 3 separate APC ablations were performed in the esophagus of the 2nd and 3rd pigs (30, 40 and 50 cm from the incisors).

### Post procedure monitoring

Post-procedure protocol included a liquid diet on day one (Ensure—3 cans three times per day), progressed to regular diet afterwards. Vital signs were monitored and recorded 3 times on post-operative day one, and then once daily. Post-operative pain was managed with carprofen (Rimadyl) as needed at 5–10 mg/kg via subcutaneous injection. In addition, potential complications such as bleeding and perforation were assessed by monitoring stool and urine outputs, oral intake, and reluctance to move.

### Validation of stricture formation

The first follow-up EGD was performed at day 14 post APC-induced mucosal ablation (Fig. 1). A second follow-up EGD was performed 2 weeks after the first (day 28 after initial endoscopy with APC induced mucosal ablation). Weight was recorded at each point to monitor for any objective change caused by strictures (Supplementary Table 1). Severe luminal stricture was defined by the inability to look beyond the stricture with a standard adult gastroscope (EG29-i10, PENTAX Medical, USA). We observed the animals for any complications, including bleeding, perforation and ulceration.

### Post procedure follow-up and serial endoscopies

During the follow-up EGD at day 14, strictures were noted and balloon dilation was performed. A Controlled Radial Expansion (CRE) wire-guided Esophageal/Pyloric/Colonic balloon dilatation catheter (Boston Scientific, USA) over a 0.035 inch guidewire and an Alliance II Integrated Inflation/Lithotripsy device were used to perform stricture dilatation. Esophageal strictures with luminal narrowing greater than 50% were managed with endoscopic balloon dilation. The strictures were progressively dilated from 7 to 9 mm using a 6–8 and an 8–10 mm dilation balloon. If there was resistance to dilation noted during the procedure, then the largest diameter achieved was recorded. At day 28 from index EGD, a second follow-up EGD was performed to re-assess the strictures (Supplementary Table 1). Contrast injection under fluoroscopy was used during the procedure to characterize the strictures.

### Tissue harvest and histological evaluation

At day 28, after the second follow-up EGD, all pigs were sacrificed. The esophagi were harvested for histopathological evaluation. Esophageal specimens were fixed in 10% formalin overnight. Paraffin embedded tissue blocks were prepared and 6 µm sections were used for histologic examination. Hematoxylin and eosin (H&E), Masson's trichrome, and Sirius Red staining were used to identify fibrosis. The stricture fibrosis scoring were assessed by a GI pathologist of Johns Hopkins Hospital.

## Supplementary Information

Supplementary Information.

## Abbreviations

EGD: Esophagogastroduodenoscopy
ESD: Endoscopic submucosal dissection
APC: Argon plasma coagulation
N/A: Not applicable
