# Supplementary material for: A patient-like swine model of gastrointestinal fibrotic strictures for advancing therapeutics
Source: Sci Rep. 2021 Jun 25;11:13344. doi: 10.1038/s41598-021-92628-8 (PMC8233336; doi:10.1038/s41598-021-92628-8)
Supplement: Supplementary file 1 — Supplementary Information. [file 41598_2021_92628_MOESM1_ESM.pdf]

Supplementary data

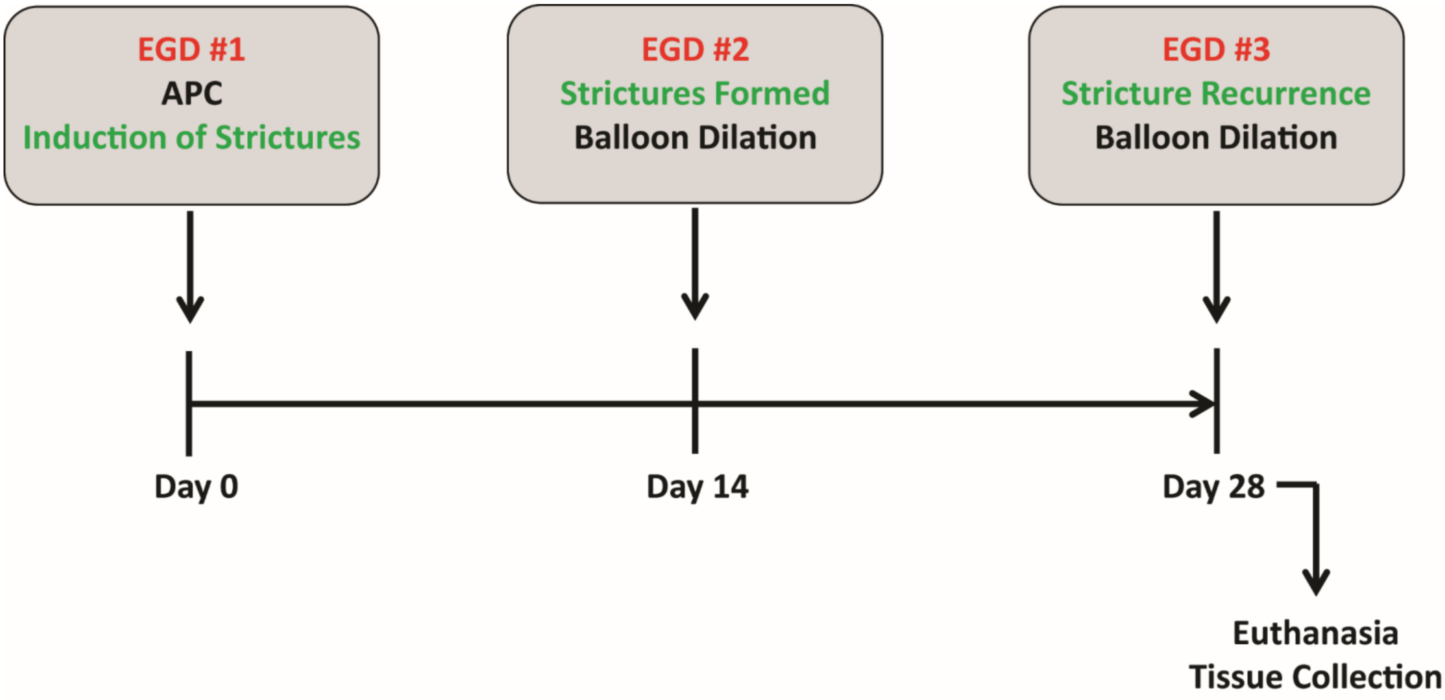

Supplementary Figure 1. Representation of the experiment. Strictures were induced at Day 0 during EGD #1. Pigs underwent EGD #2 at Day 14, at which time strictures were noted at each location where APC was applied. Strictures were dilated. Pigs underwent EGD #3 at Day 28, along with fluoroscopy, stricture dilation, and then euthanasia with tissue collection.

Supplementary Table 1. Summary Interventions Performed in the APC Induced Esophageal Pig Stricture Model

Pig ID	APC procedure to create stricture (Day 1)					Stricture(Day 14)						Stricture(Day 28)							Follow-up Day1 to Day 28
	Weight(Kg)	Lesion ID	Diameter of normal esophagus	APC mode settings	Length of APC circumferential lesion (cm)	Weight(Kg)	Length of stricture	Diameter of stricture	Ballon dilation setting	Dilation success	Complication	Weight(Kg)	length of stricture	Diameter of stricture	Stricture feature	Ballon dilation setting	Dilation success	Complication	Pig behavior
Pig 1	37.3	1a	16mm	Flow: 0.8 L/min Max. Watts: 30 Effect: 2	1 cm	38.8	1 cm	6 mm	3.0 atm, 5.5 atm, 9.0 atm(9 mm)	yes	slight tear, minor bleeding	39.7	1 cm	6 mm	smooth nontraversable	2.8 atm, 7 mm ballon	yes	slight tear, minor bleeding	bright , alert, regular diet
		1b	15 mm	Flow: 0.8 L/min Max. Watts: 40 Effect: 2	3 cm		3 cm	3 mm	3.0 atm, 5.5 atm, 9.0 atm(9 mm)	yes	big tear, minor bleeding		3 cm	2 mm	pinhole	1 atm, 1.5 atm, 2.5 atm	No, endoscopy cannot pass	big tear, minor bleeding	
Pig 2	35.2	2a	16 mm	Flow: 0.8 L/min Max. Watts: 30 Effect: 2	3 cm	35.6	1 cm	3 mm	3.0 atm, 5.5 atm, 9.0 atm(9 mm)	yes	big tear, minor bleeding	33.7	3 cm	2 mm	pinhole	[illegible] 1.5 atm, 2.5 atm and 3.3	yes	big tear, minor bleeding	At Day 18, this pig started to vomit frequently, cannot tolerate solid food, full liquid food was provided till Day 28, slight streseds out lost some weight, otherwisethe pig is alert and bright
		2b	15 mm	Flow: 0.8 L/min Max. Watts: 40 Effect: 2	3cm		3 cm	2 mm	3.0 atm, 5.5 atm, 9.0 atm(9 mm)	yes	big tear, minor bleeding		3 cm	1 mm	pinhole	1 atm, 1.5 atm, 2.5 atm and	yes	big tear, minor bleeding	
		2c	16 mm	Flow: 0.8 L/min Max. Watts: 40 Effect: 2	5 cm		5 cm	2 mm	3.0 atm, 5.5 atm, 9.0 atm(9mm)	yes	big tear, minor bleeding		5 cm	0.5 mm	pinhole	N/A	No, after dilation, the endoscopy still cannot pass	big tear, minor bleeding	
Pig 3	39.2	3a	15 mm	Flow: 0.8 L/min Max. Watts: 30 Effect: 2	3 cm	40	3 cm	4 mm	3.0 atm, 5.5 atm, 9.0 atm(9 mm)	yes	big tear, minor bleeding	39.1	3 cm	2 mm	pinhole	3 atm, 4 atm, 5 atm and 5.5 atm	yes	big tear, minor bleeding	pig showed difficult to swallow regular solid food at Day 21, but still can tolerate, food was switched half liqud and half solid food till Day 28
		3b	15 mm	Flow: 0.8 L/min Max. Watts: 40 Effect: 2	3 cm		3 cm	3 mm	3.0 atm, 5.5 atm, 9.0 atm(9 mm)	yes	big tear, minor bleeding		3 cm	1 mm	pinhole	2.5 atm, 5 atm and 5.5	yes	big tear, minor bleeding	
		3c	15 mm	Flow: 0.8 L/min Max. Watts: 40 Effect: 2	5 cm		5 cm	3 mm	3.0 atm, 5.5 atm, 9.0 atm(9 mm)	yes	big tear, minor bleeding		5 cm	1 mm	pinhole	N/A	ballon guide wire cannot pass	N/A	
